# Driving a Microswimmer with Wall-Induced Flow

**DOI:** 10.3390/mi12091025

**Published:** 2021-08-27

**Authors:** Clément Moreau, Kenta Ishimoto

**Affiliations:** Research Institute for Mathematical Sciences, Kyoto University, Kyoto 606-8502, Japan; ishimoto@kurims.kyoto-u.ac.jp

**Keywords:** microswimming, active wall, cilia carpet, controllability, optimal control

## Abstract

Active walls such as cilia and bacteria carpets generate background flows that can influence the trajectories of microswimmers moving nearby. Recent advances in artificial magnetic cilia carpets offer the potentiality to use a similar wall-generated background flow to steer bio-hybrid microrobots. In this paper, we provide some ground theoretical and numerical work assessing the viability of this novel means of swimmer guidance by setting up a simple model of a spherical swimmer in an oscillatory flow and analysing it from the control theory viewpoint. We show a property of local controllability around the reference free trajectories and investigate the bang–bang structure of the control for time-optimal trajectories, with an estimation of the minimal time for suitable objectives. By direct simulation, we have demonstrated that the wall actuation can improve the wall-following transport by nearly 50%, which can be interpreted by synchronous flow structure. Although an open-loop control with a periodic bang–bang actuation loses some robustness and effectiveness, a feedback control is found to improve its robustness and effective transport, even with hydrodynamic wall-swimmer interactions. The results shed light on the potentialities of flow control and open the way to future experiments on swimmer guidance.

## 1. Introduction

The motion of biological and artificial bodies in a fluid at microscopic scale, or microswimmers, has been a subject of growing interest in the last decades, with increasing understanding of the mechanisms at stake in order to efficiently swim at this scale and the rise of promising applications in the biomedical field, such as targeted drug delivery or non-invasive surgery performed by swimming microrobots. To serve these purposes, the need for propulsion and guidance of microdevices and biological particles is becoming increasingly prevalent.

Microswimmers seldom move in a perfectly quiescent fluid, and their motion is largely influenced by surrounding background flows. The general interactions of swimmers with background flows have been extensively considered, both experimentally and theoretically [1,2]. In particular, microswimmers often move in the presence of active biological walls generating an oscillatory flow, thanks to cilia beating in synchrony on walls [3,4] or on the body of microorganisms [5] or the collective motion of a bacterial carpet [6]. Active walls also play significant roles in reproductive processes such as peristaltic and ciliary pumping in the oviduct and are considered to promote sperm and ovum transport [7,8,9,10,11,12] and induce sperm rheotactic responses [13,14,15].

Furthermore, inspired by these examples of wall actuation in nature, the last few years have seen the emergence of artificial cilia carpet operated by magnetic fields [16,17,18,19], which open the way to practical wall-controlled guidance of both biological and artificial swimmers in the near future. The general idea of using an external flow as a means of guidance of a microswimmer has, to the best of our knowledge, only briefly been explored [20,21], in particular using a variable shear flow as a means of control [22]. It, however, constitutes a promising avenue to deal with situations where the use of more standard propelling strategies, such as chemical propulsion or direct magnetic guidance, which require active engines or magnetic components embedded into the swimmer, is not possible or suitable.

Therefore, in this paper, we will address the possibility of guiding a generic microswimmer by modulating the amplitude of an oscillatory flow generated by a neighbouring wall, formulating a simple model and analysing its properties with the help of mathematical control theory and numerical simulations. In addition to laying a formal mathematical foundation that fosters understanding of microswimming at a theoretical level, the control-theory standpoint can provide both guidance for designing microdevices and an optimal way of controlling them. Former theoretical studies of microswimmer control produced powerful results of controllability (the ability for a swimmer to reach any target from an initial position) and optimal control (finding the best possible way of reaching the target with respect to a given objective function); to cite only a few, we mention the works about the general deformable body [23,24,25,26,27]; robots made of spheres [28] or rigid segments connected by elastic joints, whether controlled by their deformation [29,30,31] or by an external magnetic field [32,33,34,35]; and more recently, an ellipsoidal swimmer controlled by a shear flow [22]. In line with these studies, the present analysis will thus seek to establish the viability of wall-generated flow control as a means of guidance, while motivating and informing future considerations of complex fluid and swimmer control problems and providing guidelines for future experimental setups.

Hence, in the following, we will first describe our model of a spherical swimmer moving in an oscillatory background flow, as proposed in [36]. Seeing it as a control system, we will move on to examining the theoretical local controllability of the swimmer around its reference trajectories, therefore warranting the soundness of searching for optimal trajectories with classical optimal control tools. We address several practical objectives and provide explicit approximate expressions for the structure of the optimal control and for the minimal time when the swimmer is guided along a direction parallel to the wall. Finally, in the last section, we move on to practical considerations, numerically exploring the advantages of feedback control and studying the influence of the interactions between the wall and the swimmer when assuming it behaves as a squirmer.

## 2. Model

**Figure 1:**
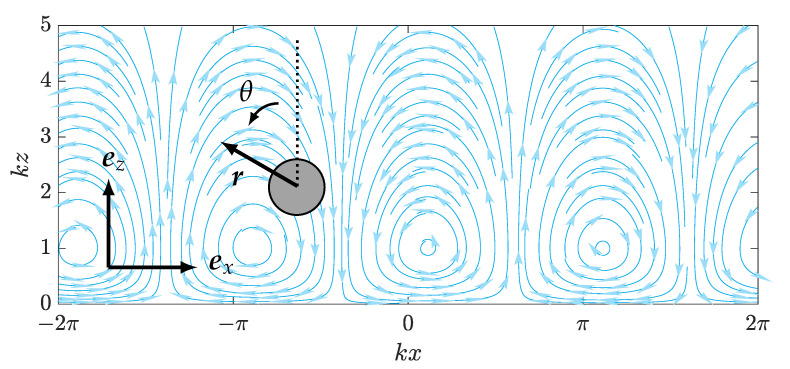
Spherical swimmer in wall-induced oscillatory flow. The swimmer moves in the xz-plane and has propulsive velocity *v* in the direction indicated by r, which makes an angle θ to the *z* axis of the laboratory frame.

We consider a spherical swimmer of radius *a*, facing a direction r with coordinates in the lab frame $\left(e_{x},e_{y},e_{z}\right)$ and swimming at a constant speed $U^{\mathrm{free}}=vr$, with v≥0. We assume that the swimmer swims in the reference plane $\left(e_{x},e_{z}\right)$, so we simply have $U^{\mathrm{free}}=\left(-v\sin\theta ,0,v\cos\theta \right)$ and no angular velocity, namely $\Omega^{\mathrm{free}}=\dot{\theta }e_{y}=0$, with θ the angle between $e_{z}$ and r—see Figure 1.

The swimmer is assumed to be swimming in the presence of a wall on z=0 that generates an oscillatory flow field of the form $u\left(t\right)\sin\left(\omega t-kx\right)e_{x}$, typically modelling a metachronal wave created by a carpet of active cilia, with u(t) being a time-varying amplitude that we will prescribe in order to influence the swimmer’s motion. Following [36], the reciprocal theorem [37] allows us to nicely calculate the effect on this flow on the swimmer’s motion:

(1) $$\begin{array}{cc} U_{x}^{\mathrm{wall}} & =u\left(t\right)e^{-kz}\left(1-kz\right)\sin\left(kx-\omega t\right), \end{array}$$

(2) $$\begin{array}{cc} U_{z}^{\mathrm{wall}} & =-u\left(t\right)e^{-kz}kz\cos\left(kx-\omega t\right), \end{array}$$

(3) $$\begin{array}{cc} \Omega_{y}^{\mathrm{wall}} & =\frac{1}{2}u\left(t\right)e^{-kz}k\sin\left(kx-\omega t\right). \end{array}$$

This wall-generated flow is represented in Figure 1, with flow cells centred at height kz and of width π/k rotating in alternate directions. Thus, the dynamics of the swimmer is governed by the following system of equations:

(4) $$\left\{\begin{array}{cc} \dot{x} & =U_{x}^{\mathrm{free}}+U_{x}^{\mathrm{wall}}\\ \dot{z} & =U_{z}^{\mathrm{free}}+U_{z}^{\mathrm{wall}}\\ \dot{\theta } & =\Omega_{y}^{\mathrm{wall}}. \end{array}\right.$$

In this system, the swimmer–wall hydrodynamic interactions may be neglected, since the wall-induced flow is significantly larger in the practical situation, which will be further discussed in the later section.

Defining the *state* $X={\left[x,z,\theta \right]}^{T}$, we will write System (4) under the generic control system form

(5) $$\dot{X}=f\left(X,u\left(t\right)\right),$$

where f encodes the dynamics in (4). The time-varying amplitude u(t), which we will refer to as the *control*, allows us to act on the swimmer’s state X, i.e., its position and orientation. The purpose of this study is to determine the properties of this system from the control theory point of view, i.e., the capacity of *u* to drive the swimmer to a required state, and how to achieve it in practice. We will start by addressing the *controllability* of system (4); in other words, the existence of controls that allow for steering it to a target state. With this theoretical analysis set up, we will then move on to finding the optimal controls for relevant objectives.

## 3. Results

### 3.1. Controllability Analysis

In this section, we examine the controllability properties of the flow-driven swimmer in a local sense around reference trajectories $X_{\mathrm{ref}}$. The idea is to answer the following question, which constitutes an important theoretical feature for a controlled system: can we target any position and orientation within a neighbourhood of the swimmer path $X_{\mathrm{ref}}$ using a control of “small” amplitude that stays close to a reference value $u_{\mathrm{ref}}$? Here, we will focus on the particular and arguably more practical situation $u_{\mathrm{ref}}=0$, therefore seeking to control the position and orientation of the free swimmer by generating a flow of “small” amplitude with the neighbouring wall.

Consider an initial state $X_{0}={\left[x_{0},z_{0},\theta_{0}\right]}^{T}$ for the swimmer, and let $X_{\mathrm{ref}}={\left[x_{\mathrm{ref}},z_{\mathrm{ref}},\theta_{\mathrm{ref}}\right]}^{T}$ be the solution of the system starting at $X_{0}$ with no flow, on the time interval [0,T], which is simply given by $X_{\mathrm{ref}}\left(t\right)={\left[x_{0}-vt\sin\theta_{0},z_{0}+vt\cos\theta_{0},\theta_{0}\right]}^{T}$: with no external flow, the swimmer swims along a straight line.

**Figure 2:**
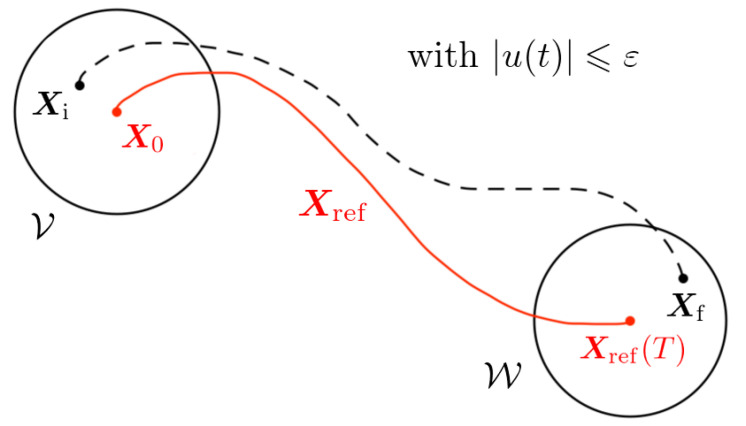
Schematic representation of local controllability around a reference trajectory $X_{\mathrm{ref}}$, in which the swimmer moves along the dashed trajectory between the neighbourhoods V and W whilst the control u(t) is kept small. Informally, the system is locally controllable around the trajectory $X_{\mathrm{ref}}$ if such a dashed trajectory joining $X_{i}$ and $X_{f}$ exists for all states $X_{i}\in V$ and $X_{f}\in W$.

Local controllability around $X_{\mathrm{ref}}$ is schematised on Figure 2 and formally defined as follows [38]: System (4) is said to be locally controllable around the trajectory $\left(X_{\mathrm{ref}}\right)$ if, for all ε>0, there exists neighbourhoods V and W of $X_{0}$ and $X_{\mathrm{ref}}\left(T\right)$ such that, for all $X_{i}$ in V, and $X_{f}$ in W, there exists a bounded control *u* defined on the time interval [0,T], satisfying |u(t)|⩽ε for all t∈[0,T], and such that the solution of System (4) starting at $X_{i}$ satisfies $X\left(T\right)=X_{f}$. Figure 2 provides a graphical illustration of this concept, with an example of controlled trajectory (in a dotted line) linking an initial state in V and a target state in W around the reference trajectory (in red).

To study the local controllability of System (4), we consider the linearised control system of System (4) around $X_{\mathrm{ref}}$, with reference control $u_{\mathrm{ref}}=0$, that reads

(6) $$\dot{X}=A\left(t\right)X+B\left(t\right)u\left(t\right),$$

with $A=\frac{\partial f(X,u)}{\partial X}\left(X_{\mathrm{ref}},0\right)$ and $B=\frac{\partial f(X,u)}{\partial u}\left(X_{\mathrm{ref}},0\right)$. It is well-known (see for instance [38] (Theorem 3.6)) that the nonlinear system is locally controllable around $X_{\mathrm{ref}}$ if the linearised system (4) is (globally) controllable. To examine if this is the case, we can apply a simple algebraic “Kalman-type” condition for time-varying linear control systems [39,40]: let $B_{1}\left(t\right)=\dot{B}-\mathrm{AB}$ and $B_{2}\left(t\right)=\dot{B_{1}}-AB_{1}$; then, System (6) is controllable in time *T* if and only if there exists t∈[0,T] such that the matrix $\left(B\left(t\right)|B_{1}\left(t\right)|B_{2}\left(t\right)\right)$ is invertible.

The determinant Δ of $\left(B\left(t\right)|B_{1}\left(t\right)|B_{2}\left(t\right)\right)$ can be obtained through straightforward calculations:

(7) $$\begin{array}{c} \Delta \left(t\right)=\frac{1}{2}e^{3k(z_{0}+tv\sin\theta_{0})}k^{3}v\omega_{c}\sin\theta_{0}\\ \;\;\times \left[\omega_{c}\left(z_{0}+tv\sin\theta_{0}\right)\cos\left(kx_{0}-\omega_{c}t\right)+v\sin\theta_{0}\sin\left(kx_{0}-\omega_{c}t\right)\right], \end{array}$$

with $\omega_{c}=\omega -kv\cos\theta_{0}$. We can see that the determinant vanishes if any of the parameters among *k*, *v*, $\omega_{c}$, or $\sin\theta_{0}$ is equal to 0. In those cases, the linearised system is not controllable. Upon further investigation, we can see that two of these cases are actually trivially not controllable, even globally: if k=0, the swimmer’s orientation θ remains always constant regardless of the control, indicating that a simple sliding motion of a wall is unable to control the swimmer, and the wave-like spatial inhomogeneity is necessary for the wall actuation, and if v=0 (passive particle), the particle can only follow a single flow line. Thus, the wave-like wall actuation is only able to control an active particle. For the two other cases $\omega_{c}=0$ (horizontal swimming speed equal to the velocity of the flow wave front) and $\sin\theta_{0}=0$ (vertical initial orientation), the controllability analysis alone does not allow for concluding with certainty that the nonlinear system (4) is not controllable. However, it suggests that these cases are unfavourable for driving the swimmer in an arbitrary direction and call for extra care when trying to steer its trajectory in practice.

If all of the above parameters are nonzero, then the determinant Δ(t) does not vanish (or, at worst, vanishes on isolated points), and therefore system (4) is locally controllable around $X_{\mathrm{ref}}$.

Overall, the controllability analysis shows that, except in a few unfavourable cases described above, the flow generated by the active wall is able to locally influence the trajectory of the swimmer, both in positional and rotational dynamics. We have therefore theoretically validated the usability of this means of control and will carry on towards constructive determination of optimally controlled trajectories.

### 3.2. Optimal Control

For practical guidance of a swimmer using the active wall flow, we will study optimal control problems minimising the time required to reach a set target. Formally, given an initial state $X_{0}={\left[x_{0},z_{0},\theta_{0}\right]}^{T}$ and a target $X_{1}$, this problem can be stated as follows:

(8) $$\left\{\begin{array}{cc} \min & T\\ s.t. & \dot{X}=f\left(X,u\right)\;\mathrm{on}\;\left[0,T\right],\\ \; & u\in [u_{\min},u_{\max}],\\ \; & X\left(0\right)=X_{0},\\ \; & X\left(T\right)=X_{1}. \end{array}\right.$$

We will focus on three situations (“Problems”) of interest that together allow for fully steering the trajectory of the swimmer:

1. A displacement parallel to the wall, from $x_{0}$ to $x_{1}$ at fixed distance $z_{0}$: $X_{1}={\left[x_{1},z_{0},*\right]}^{T}$;
2. A change of orientation, from $\theta_{0}$ to $\theta_{1}$ at fixed distance $z_{0}$: $X_{1}={\left[*,z_{0},\theta_{1}\right]}^{T}$;
3. A change of distance to the wall, from $z_{0}$ to $z_{1}$: $X_{1}={\left[*,z_{1},*\right]}^{T}$.

A star (∗) denotes the variables for which the final value is not fixed. Problem 1 is a natural goal that one would wish to address in this situation, consisting of enhancing, with the external flow, the transportation of the swimmer along the horizontal direction. Problems 2 and 3 complete this main objective by adding the capacity of steering the swimmer close to or away from the wall, addressing the practical issue of trapping a swimmer near the wall or letting it escape. Note that Problem 3 is less constrained than Problems 1 and 2, as only one variable is fixed at a final time; for that reason, it will be partly contained in Problem 2, as we will see later. As we observe in the following, the capacity to control the swimmer’s orientation via Problem 2 is also useful for guidance along the wall (Problem 1) for a long distance by occasionally helping the swimmer to face back in its goal direction.

Using classical optimal control theory, we address the time-optimal problem (8) by writing the associated Hamiltonian *H*:

(9) $$\begin{array}{cc} H & =p\;\cdot \;\dot{X}+p^{0},\\ \; & =u\left(t\right)e^{-kz}\left[\left(p_{x}\left(1-kz\right)+\frac{p_{\theta }}{2}\right)\sin\left(kx-\omega t\right)-p_{y}kz\cos\left(kx-\omega t\right)\right]\\ \; & \;+v\left(p_{y}\cos\theta -p_{x}\sin\theta \right)+p^{0}, \end{array}$$

where $p^{0}⩽0$, and $p={\left[\begin{array}{ccc} p_{x} & p_{y} & p_{\theta } \end{array}\right]}^{T}$ is non-trivial and a solution to the adjoint system $\dot{p}=\frac{\partial H}{\partial X}$. By virtue of the Pontryagin maximum principle, *u* must maximise *H* along the trajectory, from which we deduce that it switches between values $u_{\min}$ and $u_{\max}$, depending on the sign of the term between brackets in (9), which we will denote by h(p,X). Hence the optimal control is simply expressed as a so-called *bang–bang* function:

(10) $$u\left(t\right)=\left\{\begin{array}{cc} u_{\min} & \;\mathrm{if}\;h(p,X)<0,\\ u_{\max} & \;\mathrm{if}\;h(p,X)>0,\\ 0 & \;\mathrm{if}\;h(p,X)=0. \end{array}\right.$$

Note that if h(p,X) vanishes on an interval, one can quickly deduce that p=0 on this interval, which contradicts the non-triviality of p; thus, the third case of (10) is almost void here, and the control always switches between $u_{\min}$ and $u_{\max}$. This is valid for any optimal problem including the aforementioned objectives 1, 2, and 3; however, different values and constraints on $X_{0}$ and $X_{1}$ yield different dynamics for the Hamiltonian, requiring ad hoc analysis for each problem in order to determine the optimal control.

Let us now set $u_{\min}=-u_{\max}$ and focus on Problem 1: moving the swimmer from $x_{0}$ to $x_{1}>x_{0}$ in minimal time, while keeping it at a distance $z_{0}$. To further the analysis, we assume that the wave velocity ω/k is sufficiently larger than the swimmer speed *v* and, from Equation (1), the characteristic parallel flow speed at distance $z_{0}$, defined by

(11) $$V_{0}=u_{\max}e^{-kz_{0}}\left(1-kz_{0}\right),$$

and that θ is close to −π/2, so that the swimmer is moving towards the target $x_{1}$. These assumptions imply that the dynamics of the adjoint vector p, as well as those of *z* and θ, change slowly with respect to the timescale 2π/ω, so the sign changes of the bracketed term in (9) are approximately given by those of sin(kx−ωt).

In other words, to maximise the Hamiltonian on a short timescale, the flow amplitude *u* must switch between $u_{\min}$ and $u_{\max}$ every time sin(kx−ωt) changes sign, which occurs when the swimmer crosses the border between two of the flow cells generated by the wall. The time $T_{\mathrm{switch}}$ elapsed between two switches can then be estimated as the ratio between the wall wavelength $\frac{\pi }{k}$ and the difference between the wall flow velocity and swimmer effective horizontal velocity, yielding

(12) $$T_{\mathrm{switch}}=\frac{\pi }{\omega -k(v\sin\theta +v_{\mathrm{flow}})}.$$

In (12), the flow velocity term $v_{\mathrm{flow}}$ accounts for the average velocity gained by the swimmer while crossing the flow cell.

In order to estimate $v_{\mathrm{flow}}$, we consider the leading-order approximation of *z* and θ at the small timescale $T_{\mathrm{switch}}$, hence simply assuming that the swimmer remains at a constant distance from the wall $z_{0}$ and orientation $\theta_{0}$. We then consider a time interval of length $T_{\mathrm{switch}}$ in which the control is set to the constant value $u_{\max}$, which consequently allows us to explicitly solve the dynamics for *x*:

(13) $$x\left(t\right)=\frac{1}{k}\left[\omega t-2\arctan\left(\frac{\omega_{0}}{\omega }\tan\left(\frac{\omega_{0}\left(t-T_{0}\right)}{2}\right)-\frac{V_{0}k}{\omega }\right)\right]+vt\sin\theta_{0},$$

with $\omega_{0}=\sqrt{\omega^{2}-V_{0}^{2}k^{2}}$, and $V_{0}$ is defined in (11). At the lowest order, the displacement contributed by the flow between two switches is therefore given by $\Delta x=\frac{2V_{0}}{\omega }$, and the average velocity $v_{\mathrm{flow}}$ that the swimmer gains from the wall flow satisfies the equation

(14) $$v_{\mathrm{flow}}=\frac{\Delta x}{T_{\mathrm{switch}}}=\frac{2V_{0}}{\pi }\left(1-\frac{k(v\sin\theta_{0}+v_{\mathrm{flow}})}{\omega }\right),$$

and hence we obtain, at the lowest order,

(15) $$v_{\mathrm{flow}}=\frac{2V_{0}}{\pi }\left[1-\frac{k}{\omega }\left(v\sin\theta_{0}+\frac{2V_{0}}{\pi }\right)\right],\;T_{\mathrm{switch}}=\frac{\pi }{\omega -\left(v\sin\theta_{0}+\frac{2V_{0}}{\pi }\right)}.$$

Finally, the minimal time $T_{\min}$ for the swimmer to reach $x_{1}$ from $x_{0}$ can be estimated as

(16) $$T_{\min}=\frac{x_{1}-x_{0}}{v+v_{\mathrm{flow}}}.$$

**Figure 3:**
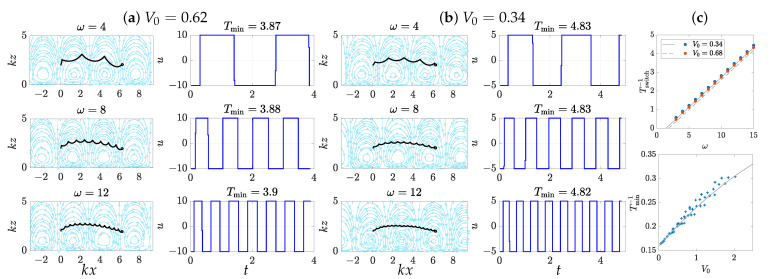
Visualisation of the time-optimal trajectories for a displacement parallel to the wall with units of k=v=1. Panels (**a**,**b**) display various trajectories obtained by solving the optimal control problem, with the swimmer going from $x_{0}=0$ to $x_{1}=2\pi /k$. On each pair of graphs, the left panel shows the path of the swimmer within the flow field, and the right panel shows the value of the control along time, with periodic switches. The trajectories for three different frequencies ω (ω=4,8,12) are displayed along with two different values of the characteristic speed $V_{0}$ (0.62 and 0.34, respectively, on panels (**a**,**b**), nondimensionalised with respect to the swimmer speed *v*). The top graph of panel (**c**) shows the relationship between the control switch time $T_{\mathrm{switch}}$ and the frequency ω. The blue and red dots correspond to numerically observed values, while the lines plot the expression obtained in Equation (15). The bottom plot shows the relationship between $V_{0}$ and the optimal time $T_{\min}$. The dots show the numerical values obtained with various values of $V_{0}$ (varying $z_{0}$ and $u_{\max}$), and the line is the prediction given by Equation (16), showing remarkable agreement.

The expressions obtained above show a linear relationship between the switch time $T_{\mathrm{switch}}^{-1}$ and the frequency ω. Moreover, for small $V_{0}$, $T_{\min}$ is minimal when $V_{0}$ is maximal, which is achieved at $z_{0}=2/k$, suggesting that this distance to the wall is optimal for parallel displacement purposes. There, setting $z_{0}=2/k$, we can give an estimation of the optimal horizontal velocity boost *b* given by the flow

(17) $$b=\frac{v_{\mathrm{flow}}}{v}=\frac{2e^{-2}}{\pi }\;\cdot \;\frac{u_{\max}}{v},$$

which amounts to approximately 8.5% if $u_{\max}=v$, and up to a boost of 43% if $u_{\max}=5v$, as in Figure 3a.

To validate this analysis, we numerically computed the optimal controls for a range of parameters, using the CasADi framework [41] and the in-built ordinary differential equation solvers in MATLAB^®^ (The Mathworks, Inc., Natick, MA, USA) [42] for the solutions of the control system (4). For this numerical assessment, we set the swimmer speed *v* and the wave number *k* to be of unit magnitude, which is equivalent to nondimensionalise the system (4) with respect to *v* and the wall wave velocity ω/k; then, in Figure 3, the value of ω, *t*, and $V_{0}$ respectively represent, in non-dimensional variables, the ratios ω/kv, tkv, and $V_{0}/v$. The range of ω is taken to match the regime in which we carried out our analysis (ω/k sufficiently larger than *v*, with a ratio of 4 seeming to be already satisfying when looking at Figure 3). Figure 3 displays the results and shows good agreement with the theory. Figure 3a,b shows the optimal trajectory and associated control function u(t) for different values of $V_{0}$ and ω, showing a characteristic aspect due to the successive switches in *u*, and confirming our prediction that the optimal time $T_{\min}$ depends very little on the frequency ω. The relationships between the switching time $T_{\mathrm{switch}}$ and ω and between the optimal time $T_{\min}$ and the characteristic speed $V_{0}$ are shown in Figure 3c, with remarkable agreement between the numerical values and the curves predicted by Equations (15) and (16). 

**Figure 4:**
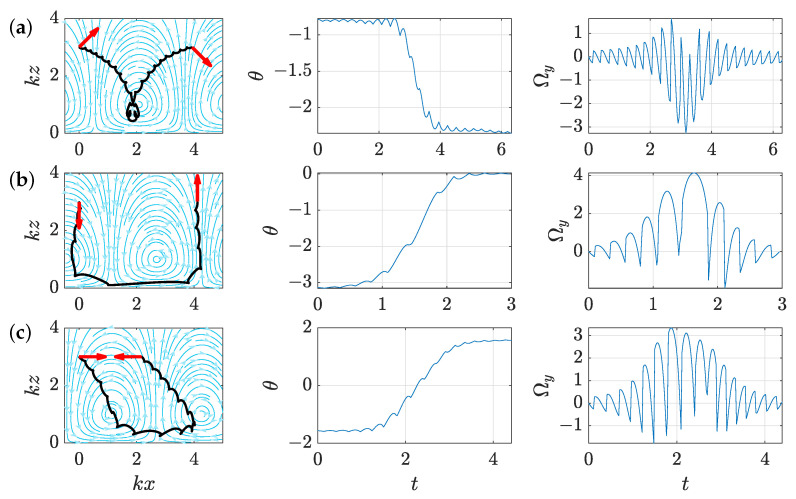
Time-optimal trajectories for changing the swimmer’s orientation with units of k=v=1 as in Figure 3. In each row is displayed an optimal trajectory from an initial orientation $\theta_{0}$ to a target orientation $\theta_{1}$, while remaining at a distance $z_{0}$ from the wall. The path followed in the xy-plane is shown on the left panel, while the evolution of the orientation θ with respect to time is shown on the middle panel, and the local rotational speed of the flow $\Omega_{y}$ with respect to time is shown on the right panel. The values of initial and target orientation $\left(\theta_{0},\theta_{1}\right)$ for rows (**a**–**c**) are respectively given by (−π/4,−3π/4), (−π,0), and (−π/2,π/2) and represented by red arrows on the trajectory plots on the left. For each case, we can distinguish three phases: approach to the wall with little variation of θ, rotation close to the wall, and going away from the wall again.

We now move on to our second control problem: changing the orientation of the swimmer from $\theta_{0}$ to $\theta_{1}$ with identical initial and final distance to the wall $z_{0}$. A few numerically computed trajectories realising this objective for various couples $\left(\theta_{0},\theta_{1}\right)$ are displayed on Figure 4. While switches in the control occur with roughly the same period $T_{\mathrm{switch}}$ as for the parallel displacement problem, in line with the short timescale analysis above, the observation of these trajectories in that case shows a more complex structure emerging for this control problem, with three distinctive phases in the swimmer’s trajectory. First, the control makes the swimmer approach the wall and then rotates it once it is close enough to the wall, before driving it away from the wall again to reach the target distance $z_{0}$. During the first and last phases, the swimmer’s orientation almost remains constant, seemingly indicating that an effective change of orientation cannot be obtained while the swimmer is far away from the wall. This can be understood by looking at the rotational component of the flow, $\Omega_{y}$ (see Equation (4)), along the optimal trajectories, plotted on the right-hand side of Figure 4. Approaching the wall allows $\Omega_{y}$ to reach large absolute values during the second phase and to generate net rotation by oscillating with non-zero average. This “approach-rotate-escape” strategy is consistently observed regardless of the initial and target orientation as well as the distance from the wall $z_{0}$. Hence, controlling the swimmer’s orientation requires efficiently controlling its distance to the wall, which is the goal of Problem 3. We can therefore conclude that the solution to Problem 3 is contained in Problem 2, with the approach and escape phases in Problem 2 corresponding to the optimal motion of the swimmer towards or away from the wall. This analysis is confirmed by numerical observations: trajectories solving Problem 3 closely resemble the ones obtained for the approach and escape phases in the trajectories solving Problem 2.

This exploration of controllability and optimal control properties of the wall-driven swimmer shows that the wall-generated flow can be expected to efficiently steer the swimmer’s trajectory, both improving the swimmer’s speed when swimming parallel to the wall and allowing it to change its orientation. However, this analysis assumes idealised control and neglects the interaction of the swimmer with the wall. In the next section, we address these issues by tackling the robustness of the control and studying the influence of added far-field wall interactions.

### 3.3. Robust Driving Strategies

The optimal control analysis conducted in the previous section showed that a bang–bang periodic control with period $T_{\mathrm{switch}}$ allows for increasing the velocity of the swimmer while driving it parallel to the wall. Thinking of the practical application of this result, one may be tempted to use this control policy in an *open-loop* fashion, i.e., without changing the policy depending on the initial or current state of the swimmer, so that *u* is given by

(18) $$u\left(t\right)=u_{\max}\mathrm{sgn}\left(\sin\left(\pi \frac{t}{T_{\mathrm{switch}}}\right)\right).$$

**Figure 5:**
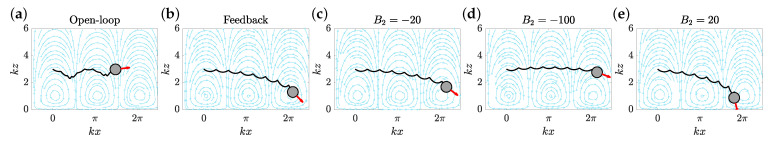
Effect of feedback control and wall interactions on the controlled trajectories. All panels show the trajectory of the swimmer starting at (0,3,−π/2) until T=5 with various parameter control policies (always with $u_{\max}=5$). The grey circle and red arrow respectively represent the swimmer and its orientation at the end of its motion. Panel (**a**) displays the trajectory obtained with the open-loop control policy defined in Equation (18), aimed at reproducing the optimal control determined in the previous section. The trajectory shows a bad synchronisation with the background flow, with little improvement of the swimmer’s average horizontal speed compared with the free swimming case. A feedback control policy is used in panel (**b**), defined as in Equation (19). The feedback helps recover synchronisation, but the swimmer’s orientation slowly changes, with risk of crashing into the wall. Panels (**c**–**e**) show the effect of the wall interaction term $B_{2}$ under the same feedback control policy. When $B_{2}<0$ (puller), as on panels (**c**,**d**), repulsion from the wall reduces the risk of crashing, all the more so the larger $|B_{2}|$ is; on the contrary, if $B_{2}>0$ (pusher), the swimmer is attracted to the wall.

This strategy has the obvious advantage of not requiring any measurement of the swimmer’s position or orientation but will typically be sensitive to measurement errors and initial conditions for that same reason. In the case of the wall-controlled swimmer, we saw in the optimal control study that the periodic control needs to be synchronised with the swimmer position, so that it switches when the swimmer reaches the border between two flow cells. If the synchronisation fails because of a small error on setting $T_{\mathrm{switch}}$, the swimmer’s motion can become far from optimal. This is illustrated by the numerical simulation displayed in Figure 5a, where the open-loop strategy is applied to the swimmer for a short time and quickly shows desynchronisation, preventing it from gaining extra velocity from the flow.

To recover better synchronisation between the swimmer and the flow, we move on from this naive open-loop strategy to a feedback (or *closed-loop*) control policy, where the flow amplitude *u* is allowed to depend on the state X. In line with the observation made in the optimal control study that the control should switch sign along with sin(kx−ωt), we then define

(19) $$u\left(t,X\right)=-u_{\max}\mathrm{sgn}\left(\sin\left(kx-\omega t\right)\right),$$

and apply this policy to drive the swimmer parallel to the wall. The resulting trajectory is displayed in Figure 5b and resembles the optimal one obtained in the previous section with good synchronisation between the flow and the swimmer and a horizontal speed gain close to the optimal one. However, one can also observe in this figure that this simple feedback strategy defined in (19) tends to rotate the swimmer towards the wall over several periods and reduce its distance to the wall, with the risk of crashing into the wall after some time. Such a phenomenon highlights the necessity of combining both strategies of parallel displacement and change of orientation to compensate for the trajectory deviations emerging even when using a feedback control.

The analysis carried on so far demonstrates the caution needed to use the optimal controls in practice and provides solid grounds for the flow guidance of microswimmers, using a simple model that neglects the influence of the swimmer’s motion on the surrounding fluid in the presence of the neighbouring wall. As briefly explained in Section 2, this is qualitatively justified by considering that the scale of these wall–swimmer interactions is typically significantly smaller than the free-swimming velocity of the swimmer, which itself is dominated by the background flow in our study; hence, the wall interactions can be neglected as a first approximation. To further validate our results, we will conclude this section by discussing the effect on the controlled trajectories when taking this interaction into account.

The wall–swimmer fluid interaction depends on the swimming strategy of the swimmer itself, which we will model from now on as a standard “squirmer” [43,44] of radius *a* with one characteristic parameter $B_{2}$.

Following [36], at the leading order (far-field approximation), the additional effect of the boundary on the squirmer’s motion is given by

(20) $$U_{x}^{b}=-\frac{3a^{2}B_{2}}{40z^{2}}\sin2\theta ,\;U_{z}^{b}=\frac{9a^{2}B_{2}}{16z^{2}}\cos2\theta ,\;\Omega_{y}^{b}=-\frac{3a^{2}B_{2}}{40z^{3}}\sin2\theta ,$$

with *a* being the radius of the swimmer. By linearity, including these effects (20) in Equation (4) is simply done by adding them together, yielding the new system

(21) $$\left\{\begin{array}{cc} \dot{x} & =U_{x}^{\mathrm{free}}+U_{x}^{\mathrm{wall}}+U_{x}^{b},\\ \dot{z} & =U_{z}^{\mathrm{free}}+U_{z}^{\mathrm{wall}}+U_{z}^{b},\\ \dot{\theta } & =\Omega_{y}^{\mathrm{wall}}+\Omega_{y}^{b}. \end{array}\right.$$

The swimmer is called a *puller*, a *pusher*, or a *neutral swimmer* if the characteristic parameter $B_{2}$ respectively satisfies $B_{2}>0$, $B_{2}<0$, or $B_{2}=0$. Note that the wall effects (20) vanish at the leading order in the case of a neutral swimmer, and we can reasonably neglect the higher order terms in the context of this study.

When $B_{2}\neq 0$, it is well known [45] by cell-wall hydrodynamic interactions that pullers face towards neighbouring walls while pushers tend to swim parallel to them. We subsequently investigated, in Figure 5c–e, the influence of this additional term in the feedback control trajectories. As expected, in the pusher case ($B_{2}=-20$), displayed in Figure 5c, the repulsive effect bends the trajectory away from the wall, although relatively weakly; a larger (and possibly unrealistic) absolute value of $B_{2}$ ($B_{2}=-100$) yields a trajectory looking almost parallel to the wall in Figure 5d. Hence, this added repulsive effect seemingly provides increased stability for the control. On the contrary, in the case of a puller $\left(B_{2}=20\right)$, the trajectory bends faster towards the wall, reducing the average horizontal speed of the swimmer and seemingly making the task of guiding it along the wall more challenging.

It is worth highlighting that the values of the parameter $B_{2}$ yielding a visible effect on the swimmer’s trajectories in Figure 5 are notably large with respect to the other parameters of the system, suggesting that the flow controls prescribed above are relatively robust with respect to the wall effect on the squirmer’s motion. This observation also corroborates, as predicted above, the choice of neglecting the boundary terms (20) for most of our theoretical study. 

## 4. Discussion

In this article, we have theoretically and numerically addressed the control and guidance of a microswimmer using the flow generated by a wall. We have described a model of a simple spherical swimmer moving within a plane in the presence of a wall, which itself generates an oscillatory flow with a variable amplitude that we control in order to influence the positional and rotational dynamics of the swimmer and steer its trajectory. Using classical tools of control theory, we have examined the local controllability of the system around reference trajectories, thus partially validating its viability as a practical means of control. Further, we studied the minimal time problem for several suitable objectives, including moving along the wall, and obtained predictive results about the structure of the optimal controls and the estimated speed boost in this situation, which are remarkably confirmed by numerical simulations.

We then observed that the open-loop control strategy applied numerically has rather low robustness to initial conditions and measurement errors, due to the high level of accuracy required in the synchronisation between the swimmer position and the control switches. To compensate for the disadvantages of open-loop control, we briefly explored the possibility of using feedback control, with improved results approaching the idealised optimal solutions.

Finally, we discussed the validity of our model by taking into account the influence of the wall at far-field in the squirmer case. With our numerical observations confirming that the influence of the wall on the swimmer’s trajectory remains modest, even for high values of the squirming parameters, we conclude that they can be safely neglected for practical purposes, though the repulsive effect of the wall on a puller may slightly help controlling it when moving parallel to the wall.

Though we have restricted this study to planar motion, our model dynamics are straightforwardly adaptable to three-dimensional motion in the presence of a wall along the (x,y)-plane by linearly superposing the two flows $u_{1}\left(t\right)\sin\left(\omega_{1}t-k_{1}x\right)e_{x}$ along the *x* direction and $u_{2}\left(t\right)\sin\left(\omega_{2}t-k_{2}y\right)e_{y}$ along the *y* direction, as well as the associated solutions for the swimmer, with a suitable set of 3D coordinates. Then, the motion in a given direction parallel to the wall should be obtained by tuning the controls $u_{1}$ and $u_{2}$ to generate flow in this direction only, hence reducing to the 2D case. With the goal of this study being to lay some simple ground guidelines for the novel idea of the guidance of swimmers by wall-induced flow, we leave further exploration of more complex, 3D-specific cases to future investigation.

In summary, in this paper, we have assessed the controllability and optimal control properties of a spherical swimmer controlled by the modulation of an oscillatory flow generated by a wall and described the key features of the optimal control for motion along the wall, notably its periodicity. We have shown that the control of a swimmer with such a setup is fully realisable and provided theoretical and practical guidelines, in particular regarding feedback control and the influence of wall interactions, aimed at calibrating future experimental work, with the hope of making efficient use of the emergent artificial cilia carpets operated by a magnetic field [16,17,18,19]. Our results on how oscillatory flows can used to boost a microswimmer’s speed could also help understand in more depth the mechanisms at stake in biological contexts featuring microorganisms swimming near active walls.

## Data Availability

The data presented in this study are openly available in the following Github repository: https://github.com/clementmoreau/wall-controlled-swimmer.
